# Supplementary material for: Myh10 deficiency leads to defective extracellular matrix remodeling and pulmonary disease
Source: Nat Commun. 2018 Nov 2;9:4600. doi: 10.1038/s41467-018-06833-7 (PMC6214918; doi:10.1038/s41467-018-06833-7)
Supplement: Supplementary file 1 — Supplementary Information [file 41467_2018_6833_MOESM1_ESM.pdf]

SUPPLEMENTARY INFORMATION

***Myh10* deficiency leads to defective extracellular matrix remodeling and pulmonary disease.**

Kim et al., 2018

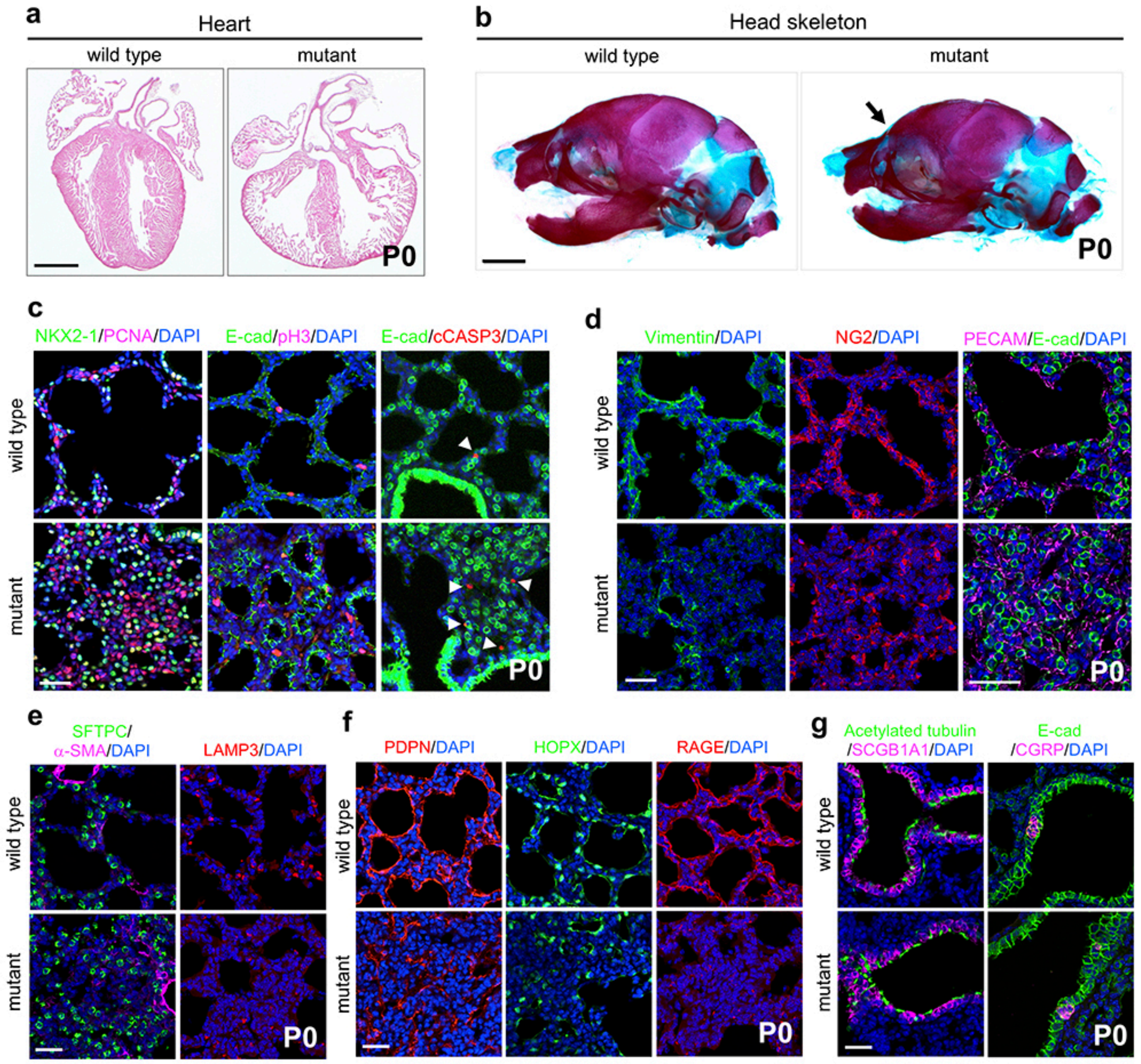

Supplementary Figure 1. Phenotypic analysis of the ENU-induced mutant. **(a)** H&E staining of the heart in wild-type (n=20) and mutant (n=20) P0 mice. **(b)** Alcian blue/Alizarin red staining of cartilage and bone in wild-type (n=8) and mutant (n=8) P0 mice. Arrow points to dome-shaped skull. **(c)** Immunostaining for NKX2-1/PCNA, E-cad/pH3, and E-cad/cCASP3 in the lung of wild-type (n=6) and mutant (n=6) P0 mice. NKX2-1 and E-cadherin mark lung epithelial cells; PCNA marks proliferating cells; Phospho-histone H3 (pH3) marks mitotic cells; Cleaved Caspase3 (cCASP3) marks apoptotic cells. Mutant lungs exhibit differentiation defects in alveolar epithelial and mesenchymal cells. **(d)** Immunostaining for Vimentin, NG2, and PECAM in the lung of wild-type (n=6) and mutant (n=6) P0 mice. Vimentin marks fibroblasts; NG2 marks pericyte-like cells; PECAM marks endothelial cells. **(e)** Immunostaining for SFTPC, α-SMA, and LAMP3 in the lung of wild-type (n=6) and mutant (n=6) P0 mice. SFTPC marks AT2 cells; α-SMA marks smooth muscle cell and myofibroblasts; LAMP3 marks mature AT2 cells. **(f)** Immunostaining for PDPN, HOPX, and RAGE in the lung of wild-type (n=6) and mutant (n=6) P0 mice. PDPN, HOPX, and RAGE mark AT1 cells. **(g)** Immunostaining for acetylated tubulin (ciliated cells), SCGB1A1 (club cells), and CGRP (neuroendocrine cells) in the lung of wild-type (n=6) and mutant (n=6) P0 mice. Scale bars: 1mm (a, b), 30 μm (c, d, e, f, g).

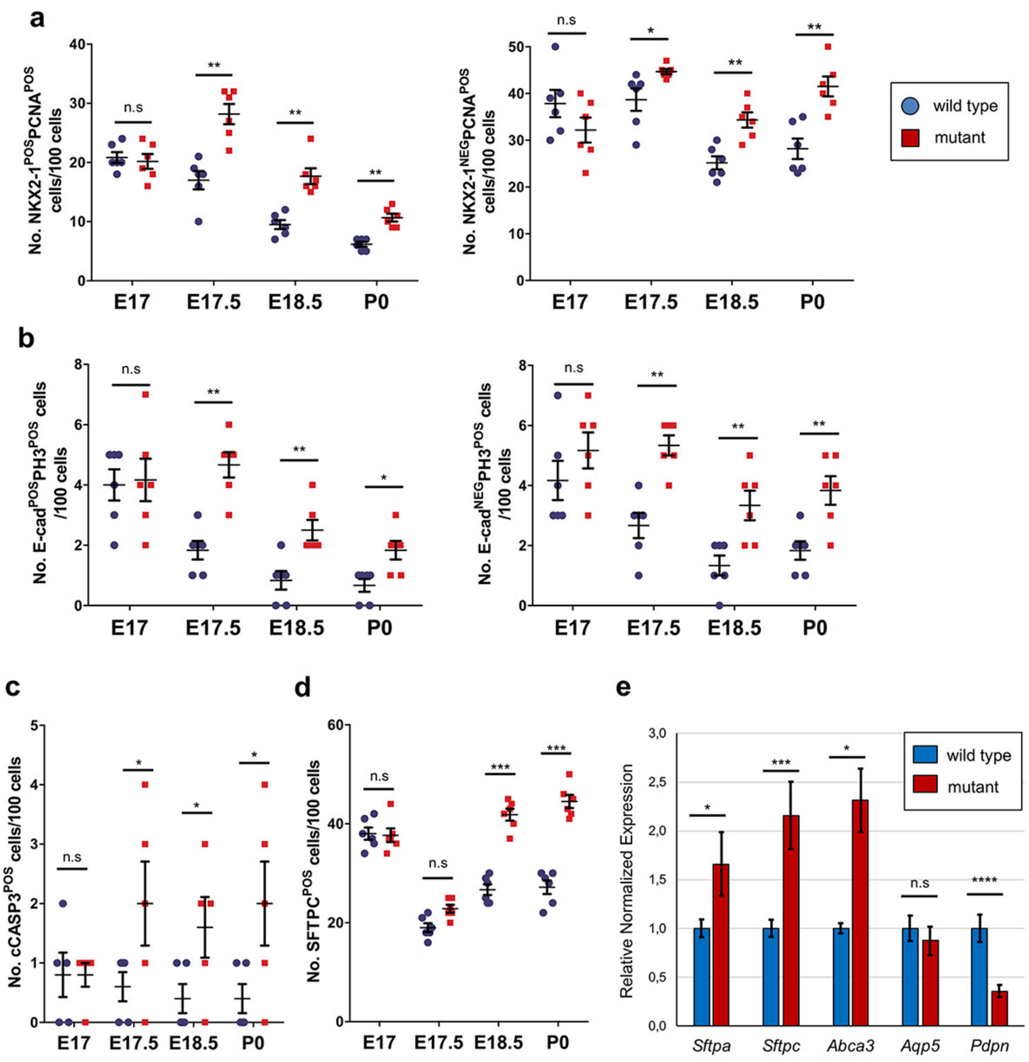

Supplementary Figure 2. Quantification of lung cell proliferation and differentiation in wild-type and mutant lungs. **(a)** Quantification of lung cell proliferation and differentiation in wild-type (n=4 for each stage) and mutant (n=4 for each stage) lungs, showing significant increases in mutants. Increased number of proliferating epithelial cells (NKX2-1/PCNA double positive cells) and mesenchymal cells (NKX2-1-negative/PCNA-positive cells) is observed in mutant lungs. **(b)** The number of mitotic epithelial cells (E-cad/pH3 double positive cells) and mesenchymal cells (E-cad-negative/pH3-positive cells) is also increased in mutants compared to wild-type siblings. **(c)** The number of apoptotic cells (cleaved CASP3-positive cells) is slightly increased in mutants compared to wild-type siblings. **(d)** The number of SFTPC-positive AT2 cells is increased in mutants compared to wild-type siblings. **(e)** Expression levels of markers of AT2 cells (*Sftpa*, *Sftpc*, and *Abca3*) and AT1 cells (*Aqp5* and *Pdpn*) in the lung of wild-type and mutant by RT-qPCR. Error bars are means ±s.e.m. n.s., not significant; *$P < 0.05$; **$P < 0.01$; ***$P < 0.001$; ****$P < 0.0001$, two-tailed Student's t test.

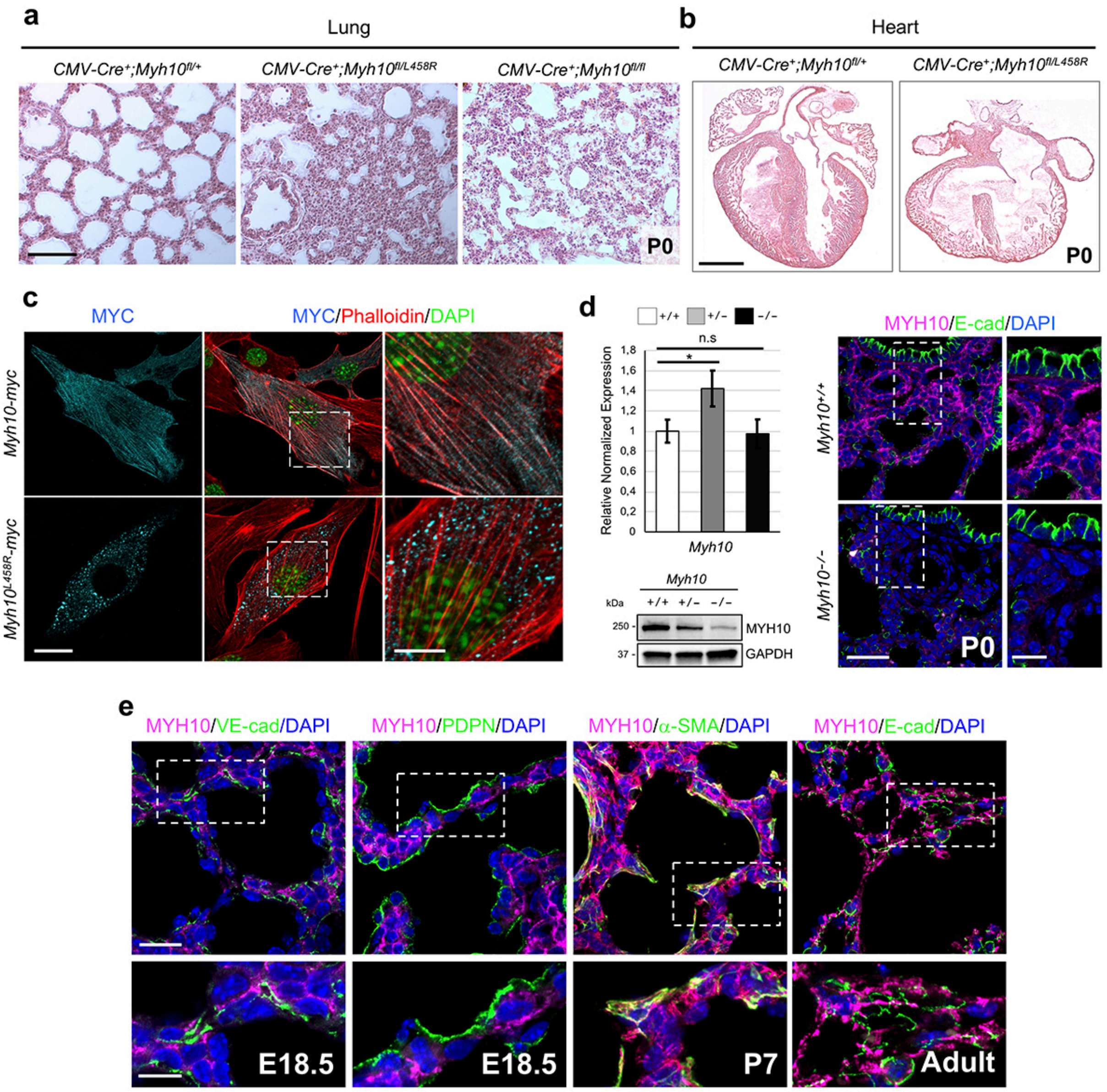

Supplementary Figure 3. Complementation test between *Myh10*^{L458R} and a *Myh10* null allele, and *Myh10* expression. **(a)** H&E staining of the lung of trans-heterozygous *Myh10*$^{L458R/+}$;*Myh10*$^{null/+}$ mice (n=5), *Myh10*^{null} mice (n=3), and wild-type littermates (n=6) at P0. **(b)** H&E staining of the heart of trans-heterozygous *Myh10*$^{L458R/+}$;*Myh10*$^{null/+}$ mice (n=5) and wild-type littermates (n=6) at P0. **(c)** Wild-type and mutant (L458R) MYH10 protein expression (from four individual sets of cultured cells) in NIH3T3 fibroblasts. MYH10-Myc is localized in scattered punctae in lamellae and co-localizes with actin in stress fibers, as visualized by phalloidin staining. The mutant protein (MYH10^{L458R}-Myc) appears dissociated from the actin bundles in stress fibers. **(d)** RT-qPCR, western blotting, and immunostaining for MYH10 (red) and E-cadherin (green) in *Myh10*$^{+/+}$ (n=15) and *Myh10*$^{-/-}$ (n=10) P0 lungs. The expression level of MYH10 is markedly reduced in *Myh10*$^{-/-}$ compared to *Myh10*$^{+/+}$ lungs. **(e)** MYH10-expressing cells do not co-express VE-cadherin (marking endothelial cells) or PDPN in E18.5 lungs. MYH10 labeling completely overlaps with α-SMA labeling in myofibroblasts of the alveolar interstitium and smooth muscle cells in P7 lungs. MYH10-expressing cells do not coincide with E-cadherin-positive epithelial cells in the adult lung. Scale bars: 1 mm (B), 50 μm (a, d (left)), 20 μm (c (left), d (right), e (top)), 10 μm (c (right)).

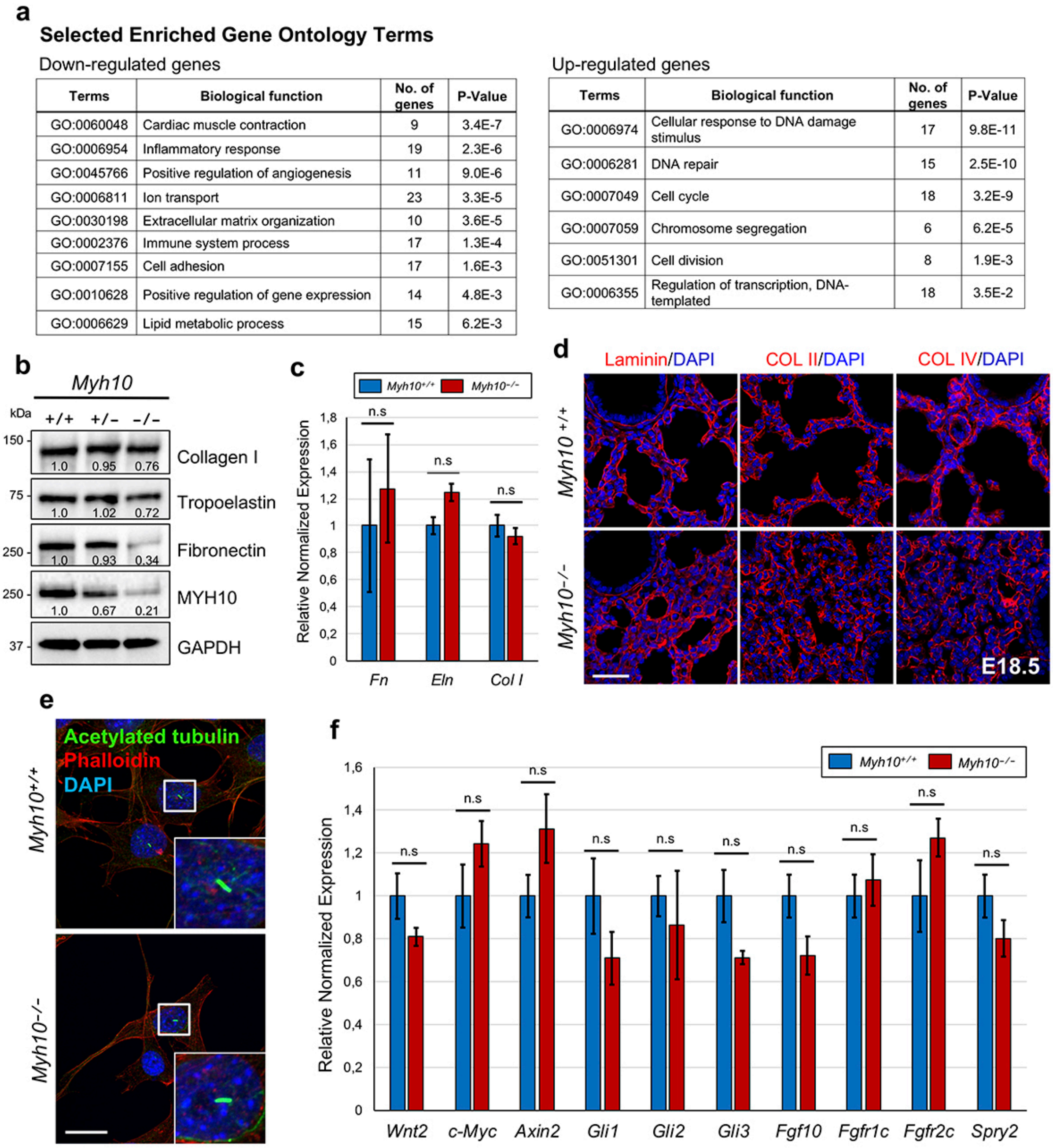

a Selected Enriched Gene Ontology Terms

Down-regulated genes

Terms	Biological function	No. of genes	P-Value
GO:0060048	Cardiac muscle contraction	9	3.4E-7
GO:0006954	Inflammatory response	19	2.3E-6
GO:0045766	Positive regulation of angiogenesis	11	9.0E-6
GO:0006811	Ion transport	23	3.3E-5
GO:0030198	Extracellular matrix organization	10	3.6E-5
GO:0002376	Immune system process	17	1.3E-4
GO:0007155	Cell adhesion	17	1.6E-3
GO:0010628	Positive regulation of gene expression	14	4.8E-3
GO:0006629	Lipid metabolic process	15	6.2E-3

Up-regulated genes

Terms	Biological function	No. of genes	P-Value
GO:0006974	Cellular response to DNA damage stimulus	17	9.8E-11
GO:0006281	DNA repair	15	2.5E-10
GO:0007049	Cell cycle	18	3.2E-9
GO:0007059	Chromosome segregation	6	6.2E-5
GO:0051301	Cell division	8	1.9E-3
GO:0006355	Regulation of transcription, DNA-templated	18	3.5E-2

Supplementary Figure 4. ***Myh10* regulates ECM remodeling.** **(a)** Gene ontology (GO) classification (all components with *p-value* <0.05 and $Log_2 < \pm 0.3$) of differentially expressed genes between *Myh10*$^{+/+}$ and *Myh10*$^{-/-}$ E17 lungs. **(b)** Representative western blots (from four individual sets of lung lysates) of ECM factor (FN, COL I, and Tropoelastin) expression in *Myh10*$^{+/+}$, *Myh10*$^{+/-}$, and *Myh10*$^{-/-}$ E18.5 lungs. Values represent the densitometric ratio of *Myh10*$^{+/-}$ and *Myh10*$^{-/-}$ to *Myh10*$^{+/+}$ after normalization to GAPDH. **(c)** Expression of *Fn*, *Eln*, and *Col I* in *Myh10*$^{+/+}$ and *Myh10*$^{-/-}$ E18.5 lungs by RT-qPCR. **(d)** Immunostaining for Laminin, Type II (COL II) and IV collagen (COL IV) on *Myh10*$^{+/+}$ (n= 10) and *Myh10*$^{-/-}$ (n=10) E18.5 lung sections. **(e)** Immunostaining for Acetylated tubulin and phalloidin of *Myh10*$^{+/+}$ and *Myh10*$^{-/-}$ fibroblasts. Acetylated tubulin marks cilia. **(f)** Expression of components of Wnt, Shh, Fgf signaling pathway in *Myh10*$^{+/+}$ and *Myh10*$^{-/-}$ E18.5 lungs by RT-qPCR. Error bars are means ±s.e.m. n.s., not significant, two-tailed Student's *t* test. Scale bars: 50 μm (c), 20 μm (e).

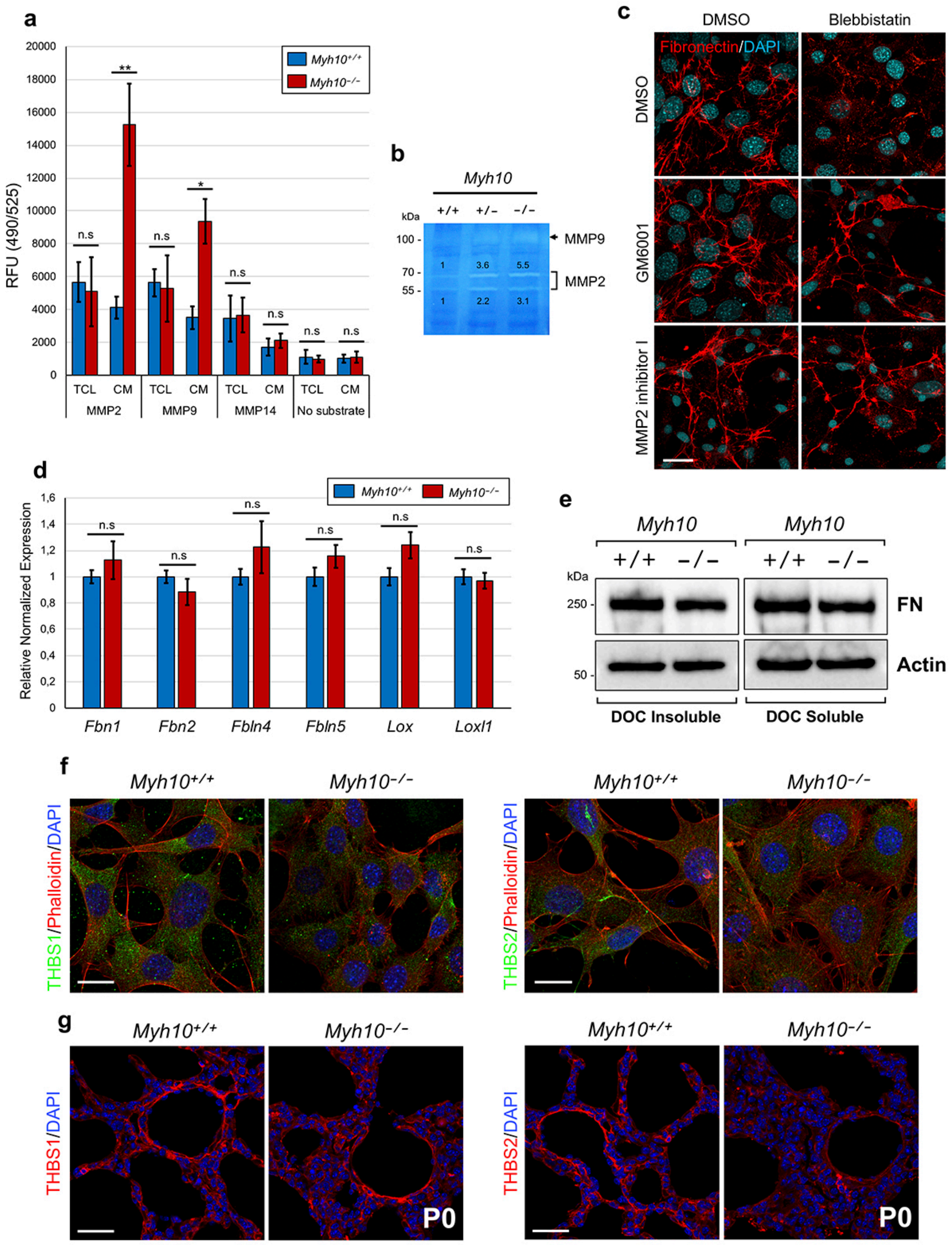

Supplementary Figure 5. ***Myh10* modulates ECM remodeling by regulating MMP and THBS proteins.** **(a)** *in vitro* MMP activity of *Myh10*$^{+/+}$ and *Myh10*$^{-/-}$ fibroblasts. CM, conditioned medium; TCL, total cell lysates. **(b)** Gelatin zymography (from three individual sets of lung lysates) of *Myh10*$^{+/+}$, *Myh10*$^{+/-}$, and *Myh10*$^{-/-}$ P0 lung lysates. Values represent the densitometric ratio of *Myh10*$^{+/-}$ and *Myh10*$^{-/-}$ to *Myh10*$^{+/+}$. **(c)** FN immunostaining (from three individual sets of cultured cells) of NIH3T3 fibroblasts treated with Blebbistatin (Bleb), GM6001, MMP2 inhibitor I, Bleb and GM6001, or Bleb and MMP2 inhibitor I. **(d)** Expression of Elastin assembly components in *Myh10*$^{+/+}$ and *Myh10*$^{-/-}$ E18.5 lungs by RT-qPCR. **(e)** Western blot analysis (from three individual sets of cultured cells) of DOC soluble and insoluble FN from *Myh10*$^{+/+}$ and *Myh10*$^{-/-}$ fibroblasts. **(f)** Immunostaining (from three individual sets of cultured cells) for THBS1 and THBS2 of *Myh10*$^{+/+}$ and *Myh10*$^{-/-}$ fibroblasts. **(g)** Immunostaining for THBS1 and THBS2 of *Myh10*$^{+/+}$ (n=8) and *Myh10*$^{-/-}$ (n=5) P0 lungs. Error bars are means ±s.e.m. n.s., not significant; $*P < 0.05$; $**P < 0.01$, two-tailed Student's t test. Scale bars: 30 μm (g), 20 μm (c, f).

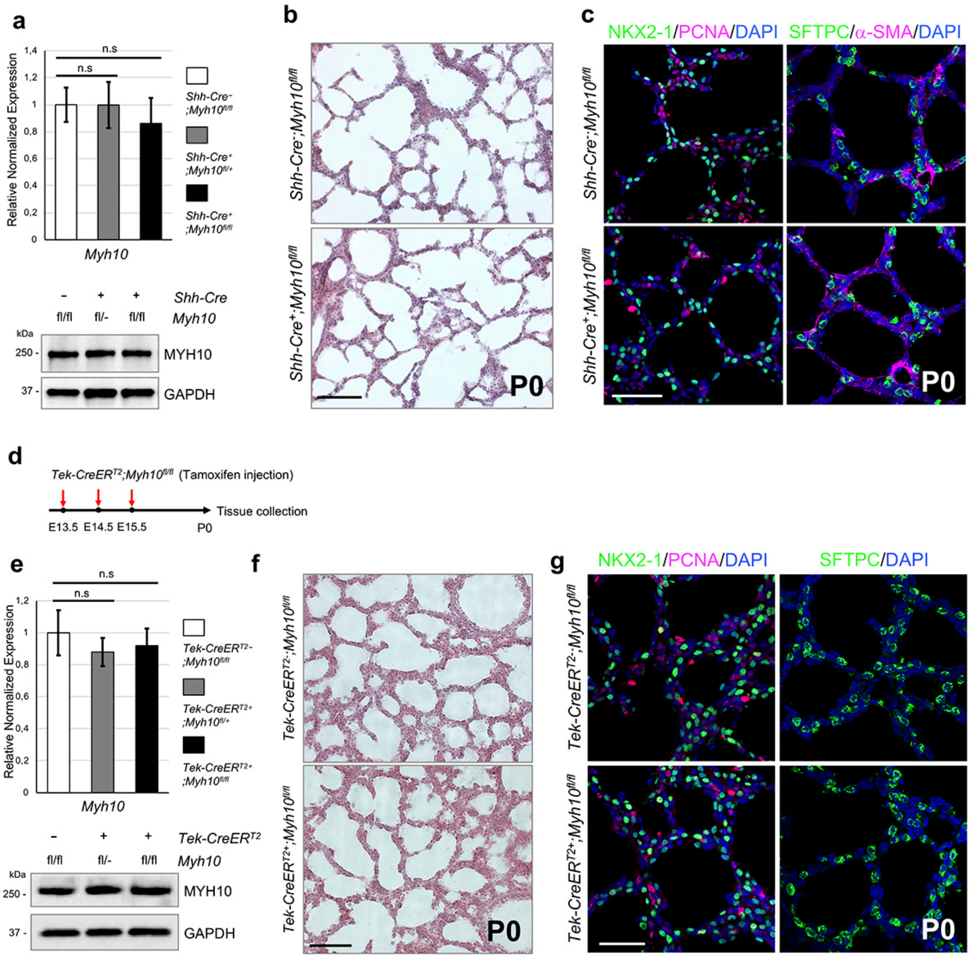

Supplementary Figure 6. Lack of lung phenotypes after epithelium- and endothelium-specific *Myh10* deletion. **(a)** *Myh10* mRNA (top) and protein (bottom) expression (from three individual sets of lung lysates) in wild-type, heterozygous, and *Shh-Cre;Myh10*^{cKO} P0 lungs by RT-qPCR and western blot, respectively. **(b)** H&E staining of wild-type (n=7) and *Shh-Cre;Myh10*^{cKO} (n=5) P0 lungs. **(c)** Immunostaining for NKX2-1/PCNA and α-SMA/SFTPC in wild-type (n=7) and *Shh-Cre;Myh10*^{cKO} (n=5) P0 lungs. **(d)** Timeline of tamoxifen (TMX) injections in *Tek-CreER*^{T2}*;Myh10*^{cKO} mice. **(e)** *Myh10* mRNA (top) and protein (bottom) expression (from three individual sets of lung lysates) in wild-type, heterozygous, and *Tek-CreER*^{T2}*;Myh10*^{cKO} P0 lungs by RT-qPCR and western blot, respectively. **(f)** H&E staining of wild-type (n=8) and *Tek-CreER*^{T2}*;Myh10*^{cKO} (n=6) P0 lungs. **(g)** Immunostaining for NKX2-1/PCNA, and SFTPC in wild-type (n=8) and *Tek-CreER*^{T2}*;Myh10*^{cKO} (n=6) P0 lungs. Error bars are means ±s.e.m. n.s., not significant; *$P < 0.05$; **$P < 0.01$, two-tailed Student's *t* test. Scale bars: 50 µm (b, f), 40 µm (c, g).

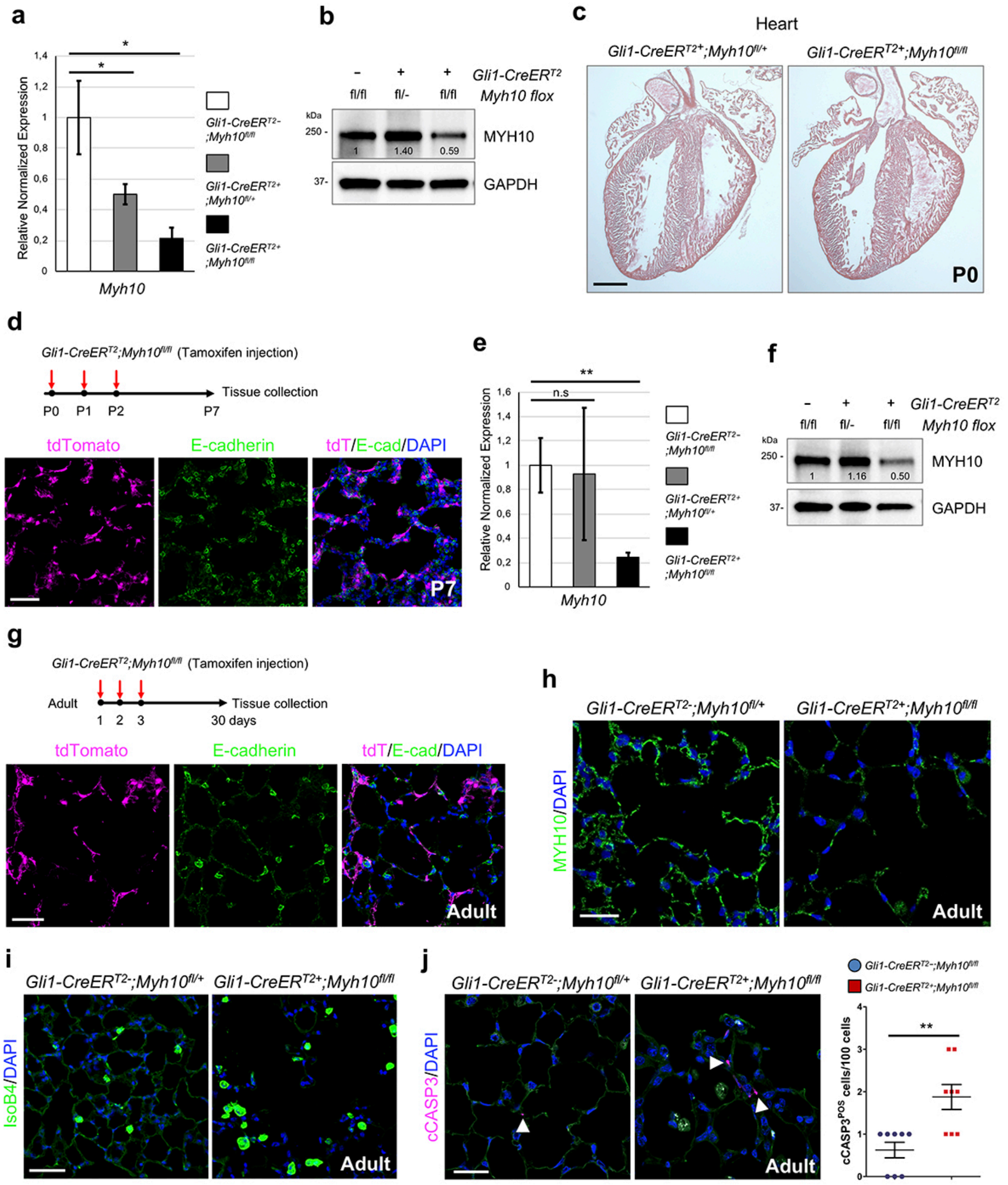

Supplementary Figure 7. Lung phenotypes after mesenchyme-specific *Myh10* deletion. **(a and b)** *Myh10* mRNA (a) and protein (b) expression (from four individual sets of lung lysates) in wild-type, heterozygous, and *Gli1-Myh10*^{cKO} P0 lungs by RT-qPCR and western blot, respectively. Values represent the densitometric ratio of *Gli1-Myh10*^{cKO} to wild type after normalization to GAPDH. **(c)** H&E staining of heart sections of wild-type (n=8) and *Gli1-Myh10*^{cKO} (n=6) P0 mice. **(d)** Diagram indicating time points of tamoxifen injections and tissue collection at postnatal stages. Immunostaining for E-caderin in *Gli1-CreER*^{T2}*;ROSA26*^{tdTomato} P7 lungs. **(e and f)** *Myh10* mRNA (e) and protein (f) expression (from four individual sets of lung lysates) in wild-type, heterozygous, and *Gli1-Myh10*^{cKO} P7 lungs by RT-qPCR and western blot, respectively. Values represent the densitometric ratio of *Gli1-Myh10*^{cKO} to wild type after normalization to GAPDH. **(g)** Diagram indicating time points of tamoxifen injections and tissue collection at the adult stage. Immunostaining for E-cadherin in *Gli1-CreER*^{T2}*;ROSA26*^{tdTomato} adult lungs. **(h)** Immunostaining for MYH10 in wild-type (n=8) and *Gli1-Myh10*^{cKO} (n=6) adult lungs. **(i)** Isolectin-FITC staining in wild-type (n=8) and *Gli1-Myh10*^{cKO} (n=6) adult lungs. **(j)** Immunostaining and quantification for cCASP3 in wild-type (n=8) and *Gli1-Myh10*^{cKO} (n=6) adult lungs. Error bars are means ±s.e.m. **P* < 0.05; ***P* < 0.01, two-tailed Student's *t* test. Scale bars: 500 μm (c), 50 (d, g), 30 μm (h, i, j).

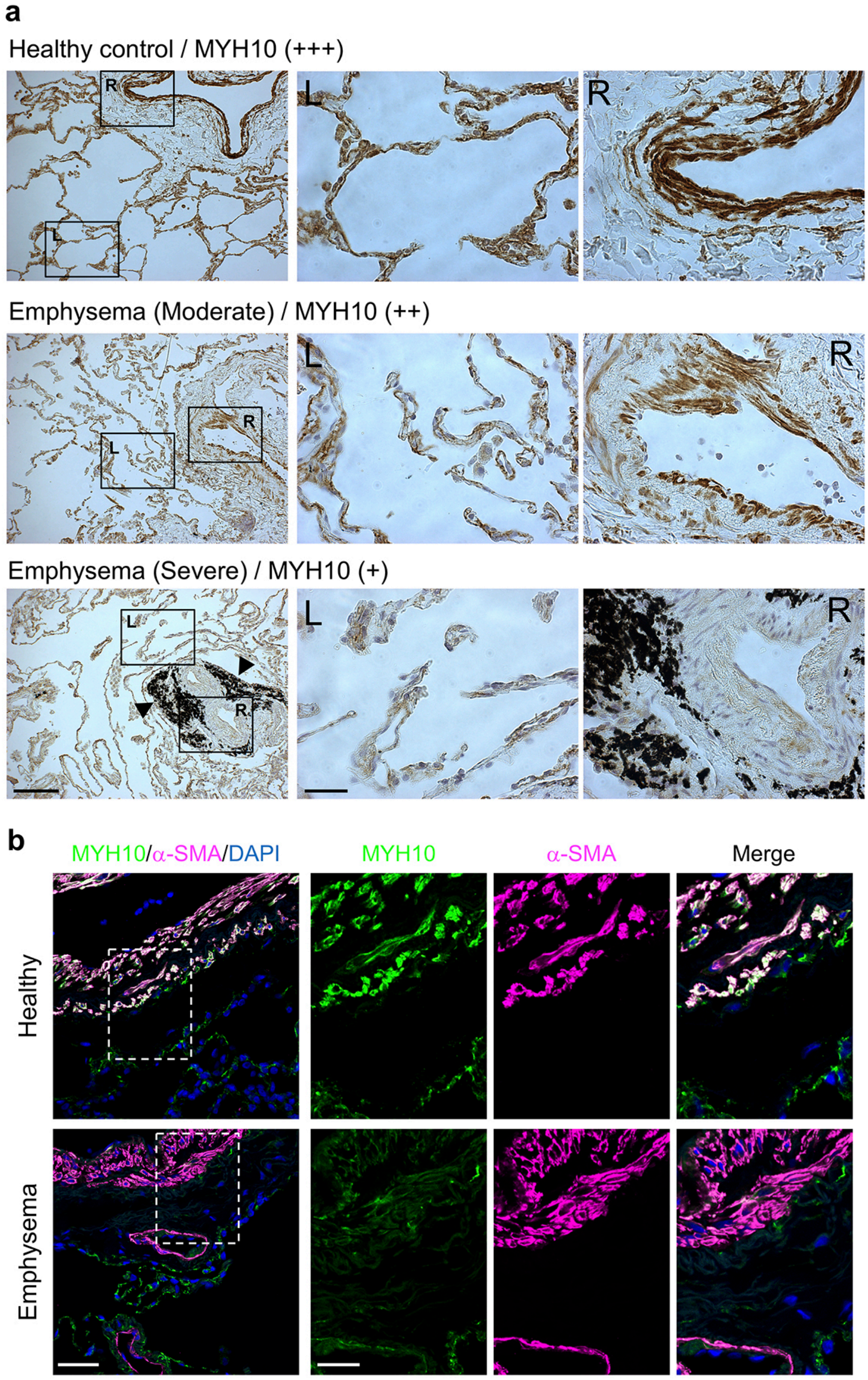

Supplementary Figure 8. MYH10 expression is downregulated in emphysematous human lungs. (a) Immunostaining for MYH10 in control and emphysematous lungs. Arrowheads point to anthracotic pigment. L, left; R, right. **(b)** Immunostaining for MYH10 and α-SMA in control and emphysematous lungs. MYH10 expression is downregulated in lung samples from emphysema patients compared to controls. α-SMA expression appears unchanged in emphysematous lungs. Scale bars: 100 μm (a (left)), 50 μm (b (left)), 25 μm (a (right), b (right)).

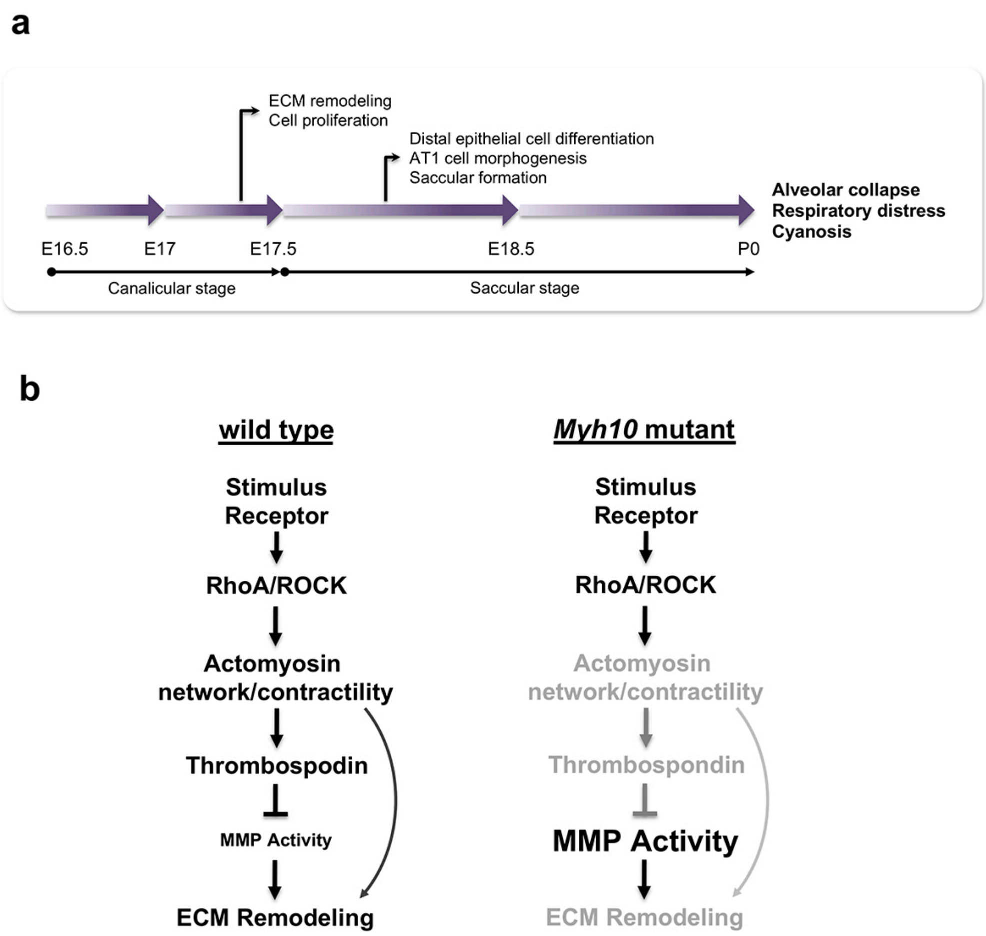

Supplementary Figure 9. Overview of the lung phenotypes and working model of *Myh10* function during lung development. **(a)** Overview of the lung phenotypes in *Myh10* mutant mice. *Myh10* mutant lungs display defects in ECM remodeling and lung cell proliferation during the canalicular stage. At the saccular stage, *Myh10* deficiency causes defects in distal epithelial cell differentiation, AT1 cell morphogenesis, and saccular formation. As a consequence, the mutant pups exhibit alveolar collapse, respiratory distress, and die shortly after birth. **(b)** Hypothetical working model of *Myh10* function during lung development. In wild types, external stimuli lead to RhoA activation and ROCK-mediated actomyosin contractility through cell surface receptors such as integrins and cadherins. In turn, increased contractility induces THBS secretion, which inhibits MMP activity, resulting in ECM remodeling. In *Myh10* deficient animals, loss of MYH10 causes defective actomyosin contractility and reduced THBS secretion, ultimately leading to MMP activation and disrupted ECM remodeling. Defective contractility of the *Myh10* mutant mesenchymal cells could also directly affect ECM assembly[56,57].

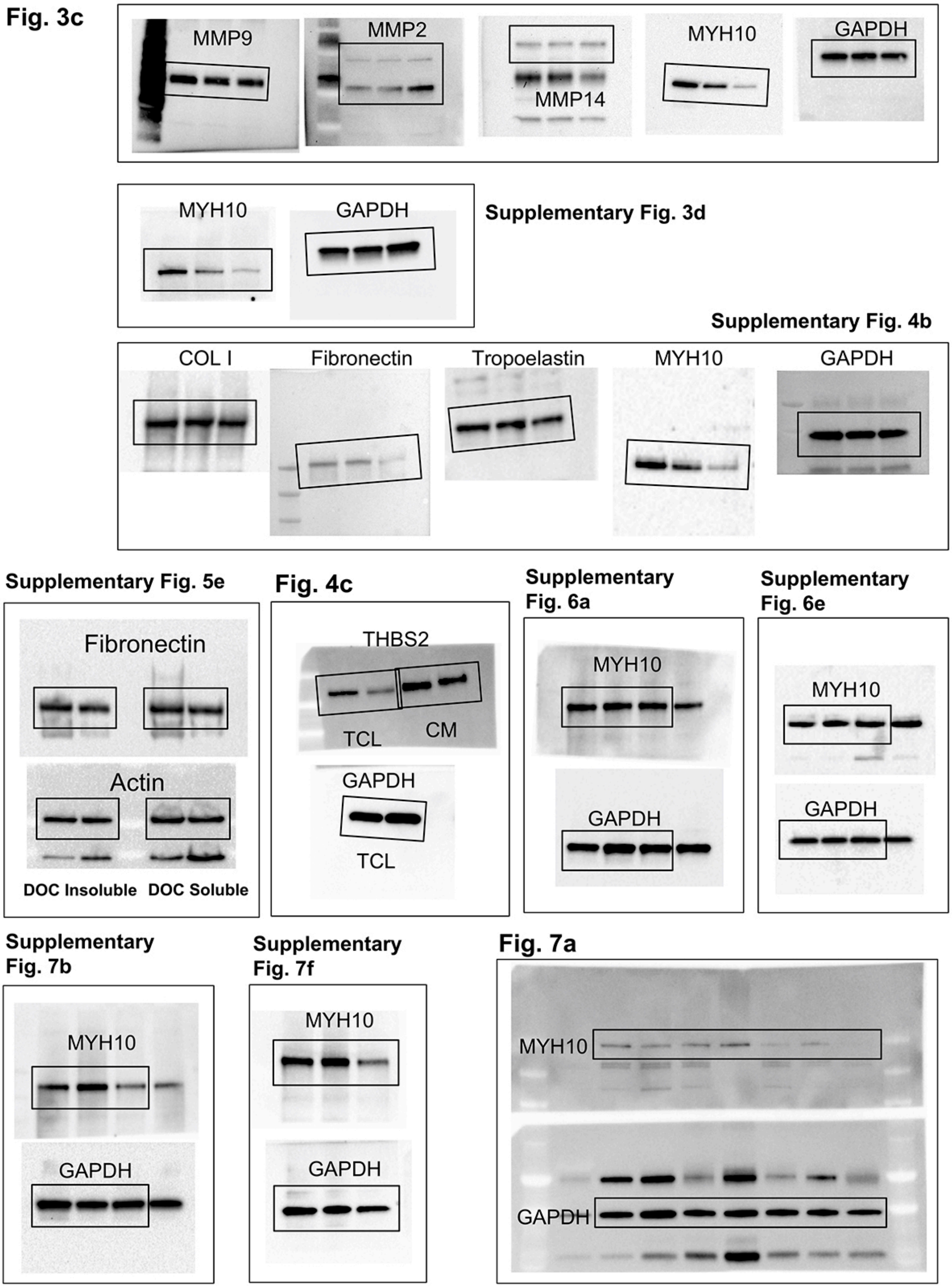

Supplementary Figure 10. Uncropped images related to western blotting data.

Supplementary Table 1. Clinical features of emphysema patients.

No.	Age (year)	Sex	Clinical Diagnosis	Severity (Emphysema group)	Smoking	IHC for MYH10
1	59	male	Donor	Emphysema grade 0 (none)	Yes	+++
2	14	female	Donor	Emphysema grade 0 (none)	No	+++
3	21	male	Donor	Emphysema grade 0 (none)	No	+++
4	16	male	Donor	Emphysema grade 0 (none)	Yes	+++
5	56	female	Donor	Emphysema grade 0 (none)	No	+++
6	34	female	Donor	Emphysema grade 0 (none)	Yes	+++
7	31	female	Donor	Emphysema grade 0 (none)	No	+++
8	43	female	COPD/Emphysema	Emphysema grade 6-7 (moderate)	No	+++
9	40	male	COPD/Emphysema	Emphysema grade 6-7 (moderate)	Yes	++
10	65	male	COPD/Emphysema	Emphysema grade 6-7 (moderate)	No	++
11	54	male	COPD/Emphysema	Emphysema grade 7-9 (severe)	Yes	+
12	55	female	COPD/Emphysema	Emphysema grade 7-9 (severe)	Yes	+
13	61	female	COPD/Emphysema	Emphysema grade 7-9 (severe)	No	+++
14	58	male	COPD/Emphysema	Emphysema grade 7-9 (severe)	Yes	++
15	59	male	COPD/Emphysema	Emphysema grade 7-9 (severe)	No	+
16	56	female	COPD/Emphysema	Emphysema grade 8-9 (severe)	Yes	++
17	57	male	COPD/Emphysema	Emphysema grade 7-9 (severe)	Yes	+++
18	59	male	COPD/Emphysema	Emphysema grade 8-9 (severe)	Yes	+
19	59	female	COPD/Emphysema	Emphysema grade 8-9 (severe)	No	++
20	55	female	COPD/Emphysema	Emphysema grade 7-9 (severe)	No	++

All patients were diagnosed with COPD/emphysema by testing pulmonary function, CT scans and pathological examination from resected lung specimens. The severity of COPD was classified according to the Global Initiative on Obstructive Lung Disease (GOLD) staging system and all subjects belonged to GOLD IV. The severity of emphysema was assessed by pathological inspection of histological samples and graded according to the method of Thurlbeck et al[69]. The expression of MYH10 is classified as follows: positive staining in <30% of cells (+); 30% to <70% of cells (++), >70% of cells (+++).

Supplementary Table 2. Sequences of primers used for RT-qPCR analysis.

Gene name	Forward primer (5'-3')	Reverse primer (5'-3')
ActB	CTCTGGCTCCTAGCACCATGAAGA	GTAAAACGCAGCTCAGTAACAGT
GAPDH	TGTGTCCGTCGTGGATCTGA	CCTGCTTCACCACCTTCTTGAT
Abca3	CAGCTCACCCTCCTACTCTG	ACTGGATCTTCAAGCGAAGCC
Aqp5	ATGAACCCAGCCCGATCTTT	ACGATCGGTCCTACCCAGAAG
Axin2	ATGTCCTGTCTGCCAGCGTTC	CAAGCACTAGCCAGTGGGTCAA
Fbn1	CAGGCTCTTCTGTGTCGATATT	TGGCTGACAGCTACATTCATAG
Fbn2	GGAGTATCGCTGTCTCTGTTATG	GATTCAGGTCACACTCGTTCA
Fbln4	CATAACCTTCCTGGCTCCTAC	GCGGTAACGACACTCATCTAT
Flbn5	TGTCAACACCTATGGCTCTTTC	GAAGCTGCACTCGTCCATATC
Fgf10	GCAGGCAAATGTATGTGGCAT	ATGTTTGGATCGTCATGGGGA
Fgfr1c	CCGTATGTCCAGATCCTGAAGA	GATAGAGTTACCCGCCAAGCA
Fgfr2c	GCCCTACCTCAAGGTTCTGAAAG	GATAGAATTACCCGCCAAGCA
Gli1	TTATGGAGCAGCCAGAGAGA	GAGCCCGCTTCTTTGTTAAT
Gli2	TGAAGGATTCCTGCTCGTG	GAAGTTTTCCAGGACAGAACCA
Gli3	AAGCGGTCCAAGATCAAGC	TGTTCCTTCCGGCTGTTC
Lox	CAAGGGACATCGGACTTCTTAC	TGGCATCAAGCAGGTCATAG
Loxl1	CTATGACCTCCGAGTGCTATTG	TCGTCCATGCTGTGGTAATG
Mmp2	AGCGTGAAGTTTGGAAGCAT	CACATCCTTCACCTGGTGTG
Myh10	CCTTCTGTTTACAATGGCCC	GTGGACAGGGCTGTCATCTA
Pdpn	ACCGTGCCAGTGTTGTTCTG	AGCACCTGTGGTTGTTATTTGT
Sftpa	GAGGAGCTTCAGACTGCACTC	AGACTTTATCCCCCACTGACAG
Sftpc	ATGGACATGAGTAGCAAAGAGGT	CACGATGAGAAGGCGTTTGAG
Spry2	GAGAGGGGTTGGTGCAAAG	CTCATCAGGTCTTGGCAGT
Wnt2	CCAACGAAAAATGACCTCGT	GGGAAGTCAAGTTGCACACA

Supplementary Table 3. RNA expression levels were analyzed by RT-qPCR.

Fig. 3f

Sample Name	Assay Name	Ct mean
EGFP siRNA, 0 nM	*GAPDH*	21.16
EGFP siRNA, 20 nM	*GAPDH*	21.76
EGFP siRNA, 100 nM	*GAPDH*	21.09
EGFP siRNA, 200 nM	*GAPDH*	21.36
Mmp2 siRNA, 0 nM	*GAPDH*	20.77
Mmp2 siRNA, 20 nM	*GAPDH*	23.96
Mmp2 siRNA, 100 nM	*GAPDH*	20.53
Mmp2 siRNA, 200 nM	*GAPDH*	20.86
EGFP siRNA, 0 nM	*Mmp2*	23.03
EGFP siRNA, 20 nM	*Mmp2*	23.54
EGFP siRNA, 100 nM	*Mmp2*	23.06
EGFP siRNA, 200 nM	*Mmp2*	23.08
Mmp2 siRNA, 0 nM	*Mmp2*	22.32
Mmp2 siRNA, 20 nM	*Mmp2*	26.09
Mmp2 siRNA, 100 nM	*Mmp2*	23.22
Mmp2 siRNA, 200 nM	*Mmp2*	23.32

Supplementary Fig. 4f

Sample Name	Assay Name	Ct mean
Myh10$^{+/+}$	*Wnt2*	28.43
Myh10$^{-/-}$	*Wnt2*	29.16
Myh10$^{+/+}$	*c-Myc*	29.77
Myh10$^{-/-}$	*c-Myc*	29.29
Myh10$^{+/+}$	*Axin2*	27.82
Myh10$^{-/-}$	*Axin2*	27.85
Myh10$^{+/+}$	*Gli1*	33.65
Myh10$^{-/-}$	*Gli1*	34.57
Myh10$^{+/+}$	*Gli2*	33.78
Myh10$^{-/-}$	*Gli2*	34.42
Myh10$^{+/+}$	*Gli3*	32.80
Myh10$^{-/-}$	*Gli3*	33.70
Myh10$^{+/+}$	*Fgf10*	20.95
Myh10$^{-/-}$	*Fgf10*	31.85
Myh10$^{+/+}$	*Fgfr1c*	29.17
Myh10$^{-/-}$	*Fgfr1c*	29.49
Myh10$^{+/+}$	*Fgfr2c*	30.94
Myh10$^{-/-}$	*Fgfr2c*	31.02
Myh10$^{+/+}$	*Spry2*	31.93
Myh10$^{-/-}$	*Spry2*	32.68
Myh10$^{+/+}$	*ActB*	22.31
Myh10$^{-/-}$	*ActB*	22.74

Supplementary Fig. 2e

Sample Name	Assay Name	Ct mean
control	*Sftpa*	19.66
mutant	*Sftpa*	20.41
control	*Sftpc*	16.27
mutant	*Sftpc*	16.63
control	*Abca3*	26.39
mutant	*Abca3*	26.65
control	*Aqp5*	24.16
mutant	*Aqp5*	23.92
control	*Pdpn*	27.23
mutant	*Pdpn*	25.71
control	*ActB*	20.35
mutant	*ActB*	19.50

Supplementary Fig. 5d

Sample Name	Assay Name	Ct mean
Myh10$^{+/+}$	*Fbn1*	27.88
Myh10$^{-/-}$	*Fbn1*	28.65
Myh10$^{+/+}$	*Fbn2*	30.57
Myh10$^{-/-}$	*Fbn2*	31.69
Myh10$^{+/+}$	*Fbln4*	27.11
Myh10$^{-/-}$	*Fbln4*	27.76
Myh10$^{+/+}$	*Fbln5*	25.34
Myh10$^{-/-}$	*Fbln5*	26.07
Myh10$^{+/+}$	*Lox*	24.51
Myh10$^{-/-}$	*Lox*	24.58
Myh10$^{+/+}$	*Loxl1*	25.22
Myh10$^{-/-}$	*Loxl1*	26.20
Myh10$^{+/+}$	*ActB*	20.59
Myh10$^{-/-}$	*ActB*	21.53

Supplementary Fig. 7a

Sample Name	Assay Name	Ct mean
Gli1-CreER^{T2-};*Myh10*$^{fl/fl}$	*Myh10*	38.22
Gli1-CreER^{T2+};*Myh10*$^{fl/+}$	*Myh10*	37.99
Gli1-CreER^{T2+};*Myh10*$^{fl/fl}$	*Myh10*	38.18
Gli1-CreER^{T2-};*Myh10*$^{fl/fl}$	*ActB*	22.28
Gli1-CreER^{T2+};*Myh10*$^{fl/+}$	*ActB*	20.36
Gli1-CreER^{T2+};*Myh10*$^{fl/fl}$	*ActB*	19.47

Supplementary Fig. 3d

Sample Name	Assay Name	Ct mean
Myh10$^{+/+}$	*Myh10*	26.15
Myh10$^{+/-}$	*Myh10*	25.85
Myh10$^{-/-}$	*Myh10*	26.37
Myh10$^{+/+}$	*ActB*	19.35
Myh10$^{+/-}$	*ActB*	20.13
Myh10$^{-/-}$	*ActB*	20.49

Supplementary Fig. 4c

Sample Name	Assay Name	Ct mean
Myh10$^{+/+}$	*Fn*	23.69
Myh10$^{-/-}$	*Fn*	23.98
Myh10$^{+/+}$	*Eln*	20.23
Myh10$^{-/-}$	*Eln*	20.85
Myh10$^{+/+}$	*Col I*	25.16
Myh10$^{-/-}$	*Col I*	26.21
Myh10$^{+/+}$	*ActB*	20.59
Myh10$^{-/-}$	*ActB*	21.53

Supplementary Fig. 6a

Sample Name	Assay Name	Ct mean
Shh-Cre^{-};*Myh10*$^{fl/fl}$	*Myh10*	32.96
Shh-Cre^{-};*Myh10*$^{fl/+}$	*Myh10*	33.20
Shh-Cre^{+};*Myh10*$^{fl/fl}$	*Myh10*	32.68
Shh-Cre^{-};*Myh10*$^{fl/fl}$	*ActB*	18.08
Shh-Cre^{-};*Myh10*$^{fl/+}$	*ActB*	17.78
Shh-Cre^{+};*Myh10*$^{fl/fl}$	*ActB*	17.21

Supplementary Fig. 6e

Sample Name	Assay Name	Ct mean
Tek-CreER^{T2-};*Myh10*$^{fl/fl}$	*Myh10*	29.15
Tek-CreER^{T2+};*Myh10*$^{fl/+}$	*Myh10*	29.98
Tek-CreER^{T2+};*Myh10*$^{fl/fl}$	*Myh10*	28.99
Tek-CreER^{T2-};*Myh10*$^{fl/fl}$	*ActB*	21.85
Tek-CreER^{T2+};*Myh10*$^{fl/+}$	*ActB*	22.47
Tek-CreER^{T2+};*Myh10*$^{fl/fl}$	*ActB*	21.25

Supplementary Fig. 7e

Sample Name	Assay Name	Ct mean
Gli1-CreER^{T2-};*Myh10*$^{fl/fl}$	*Myh10*	34.86
Gli1-CreER^{T2+};*Myh10*$^{fl/+}$	*Myh10*	35.70
Gli1-CreER^{T2+};*Myh10*$^{fl/fl}$	*Myh10*	35.89
Gli1-CreER^{T2-};*Myh10*$^{fl/fl}$	*ActB*	20.74
Gli1-CreER^{T2+};*Myh10*$^{fl/+}$	*ActB*	21.20
Gli1-CreER^{T2+};*Myh10*$^{fl/fl}$	*ActB*	22.40
