# Supplementary material for: Myh10 deficiency leads to defective extracellular matrix remodeling and pulmonary disease
Source: Nat Commun. 2018 Nov 2;9:4600. doi: 10.1038/s41467-018-06833-7 (PMC6214918; doi:10.1038/s41467-018-06833-7)
Supplement: Supplementary file 5 — Description of Additional Supplementary Files [file 41467_2018_6833_MOESM5_ESM.docx]

**Title:** Supplementary Movie 1.
**Description:** Myh10 mutant mice exhibit acute respiratory failure upon birth. P0 mutant pups exhibit respiratory distress and cyanosis.

**Title:** Supplementary Dataset 1.
**Description:** Raw data and GO terms enrichment for RNAseq analysis between E17 Myh10+/+ and Myh10-/- lungs.

**Title:** Supplementary Dataset 2.
**Description:** Raw data and GO terms enrichment for proteomics analysis between Myh10+/+ and Myh10-/- fibroblasts.
